# A Hybrid Feature Model and Deep-Learning-Based Bearing Fault Diagnosis

**DOI:** 10.3390/s17122876

**Published:** 2017-12-11

**Authors:** Muhammad Sohaib, Cheol-Hong Kim, Jong-Myon Kim

**Affiliations:** 1 Department of Electrical, Electronics and Computer Engineering, University of Ulsan, Ulsan 44610, Korea; md.sohaibdurrani@gmail.com; 2 School of Electronics and Computer Engineering, Chonnam National University, Gwangju 61186, Korea; chkim22@chonnam.ac.kr

**Keywords:** autoencoders, bearing fault diagnosis, fault diagnosis, fault severity, hybrid features, multi crack size, stacked autoencoders

## Abstract

Bearing fault diagnosis is imperative for the maintenance, reliability, and durability of rotary machines. It can reduce economical losses by eliminating unexpected downtime in industry due to failure of rotary machines. Though widely investigated in the past couple of decades, continued advancement is still desirable to improve upon existing fault diagnosis techniques. Vibration acceleration signals collected from machine bearings exhibit nonstationary behavior due to variable working conditions and multiple fault severities. In the current work, a two-layered bearing fault diagnosis scheme is proposed for the identification of fault pattern and crack size for a given fault type. A hybrid feature pool is used in combination with sparse stacked autoencoder (SAE)-based deep neural networks (DNNs) to perform effective diagnosis of bearing faults of multiple severities. The hybrid feature pool can extract more discriminating information from the raw vibration signals, to overcome the nonstationary behavior of the signals caused by multiple crack sizes. More discriminating information helps the subsequent classifier to effectively classify data into the respective classes. The results indicate that the proposed scheme provides satisfactory performance in diagnosing bearing defects of multiple severities. Moreover, the results also demonstrate that the proposed model outperforms other state-of-the-art algorithms, i.e., support vector machines (SVMs) and backpropagation neural networks (BPNNs).

## 1. Introduction

In the case of rotating machines, bearings are vital and common parts of the machine systems that are used in a variety of industries [1]. Because these parts are extensively used, bearings are prone to health degradation, which contributes to approximately 50% of the failures in electrical machines [2,3,4]. The health degradation of bearings results in unexpected failures of machines, which can lead to long downtimes, large economic losses, and human injuries [5,6,7]. Such issues can be mitigated with the help of fault diagnosis that assures the smooth operation of the systems by predicting their health states [8,9,10,11]. Bearing fault diagnosis, with the help of data obtained via vibration signals, acoustic emissions, electric currents, and temperature monitoring, has been a key area of research over the last few decades [12,13,14]. Bearing fault diagnosis is helpful in reducing the operational and maintenance costs and enhancing the reliability of a machine [15,16,17,18,19,20,21]. Vibration acceleration signals, which can be collected with an accelerometer, are extensively used in bearing fault diagnosis. Defective bearings add weak fault signatures to vibration signals whenever a rolling element strikes the fault location and can be explored via suitable signal processing techniques such as envelope analysis [22]. In general, a fault diagnosis pipeline has three stages: data acquisition, feature extraction, and fault type classification. Most recent studies related to bearing fault diagnosis have focused on identifying appropriate features of the raw vibration signals. The signals measured from the operational bearings are nonstationary and nonlinear in nature due to the variable operating conditions and multiple fault severities. Therefore, in such conditions, analysis of the measured signals by means of classical signal processing techniques alone, like the fast Fourier transform, is considered to be insufficient because they provide a global transformation that is unable to properly capture the local time–frequency properties of a signal [23]. The nonstationary behavior can be explored by various time–frequency analysis techniques, including the Wigner Ville distribution (WVD) [24], short time Fourier transform (STFT) [25,26,27], and wavelet packet transform (WPT) [28,29]. The WPT is more practical in fault diagnosis schemes because of its better time–frequency resolution. Numerous studies investigating the time domain, frequency domain, and time–frequency domain features have been carried out to design fault diagnosis schemes using vibration signals in collaboration with machine learning (ML) methods (e.g., regression models, support vector machines, and artificial neural networks (ANNs)) [30,31,32,33,34,35,36]. Huo et al. [37] presented a multi-speed fault diagnosis scheme with the help of self-adaptive wavelet transform components. Particle swarm optimization (PSO) and Broyden–Fletcher–Goldfarb–Shanno (BFGS)-based quasi-Newton minimization algorithms were considered in their scheme. The aim of their work was to determine the optimal parameters for impulse modeling the continuous wavelet transform (IMCWT). The scheme could discriminate signatures for four different health conditions. In [38], time-domain (TD) statistical features were preprocessed instead of preprocessing the vibration signal prior to implementing a classifier. Preprocessing the features helped in removing the effects of possible fluctuation and random impulses in the vibration signals. An advantage of feature preprocessing, in contrast to the traditional approach where the raw vibrational signal is preprocessed, is its computational efficiency. To achieve enhanced dimensionality reduction and improve the fault diagnosis performance, an improved manifold learning scheme based on the Mahalanobis distance (MD) was proposed in [39]. Time and frequency domain analyses were performed in the scheme to construct a high-dimensional feature set. The results of the proposed scheme were found to be better than those of the traditional manifold algorithms. The authors in [40] presented a frequency domain analysis of low-speed bearings by employing time varying and multiresolution envelope analysis (TVMREA) in combination with genetic algorithm (GA)-based discriminative feature analysis (GADFA). The proposed method effectively identified combined faults in low-speed bearings.

In recent years, deep learning has made a remarkable impact on pattern recognition, image processing, and natural language processing. Deep learning mimics the learning process of the human brain in artificial networks and has displayed superior ability in capturing useful information from the input data via non-linear transformations. In contrast to conventional machine learning algorithms, deep networks can extract highly representative features via multiple layered architectures, simplifying the learning task. In addition, deep networks keep only the most representative information in each layer and discard the rest, thereby reducing the dimensionality. Hence, due to the simplified learning capability and built-in feature reduction mechanisms, deep networks can be used for fault diagnosis of complex rotary machine bearings.

Despite the existence of several state-of-the-art bearing fault diagnosis schemes, there is still room for improvement in machine fault diagnosis; for instance, dealing with the bearing signals that exhibit nonstationary behavior due to variable working conditions and multiple fault severities. Fault pattern identification and crack size identification are two key aspects of bearing fault diagnosis. Fault pattern identification is essential as it can allow the localization of faults on a given component, whereas determining the fault severity is vital because it can highlight the urgency of repairing or replacing a damaged component. A fault diagnosis scheme that can perform both fault pattern identification and fault severity classification can be very challenging; it requires better feature representation and a strong classifier. Existing fault diagnosis schemes are vulnerable to fault misidentification due to the presence of fluctuations and random amplitudes in the vibration signals caused by multiple crack sizes.

To solve this issue, we present a two-layered fault diagnosis scheme that uses a set of hybrid features and a sparse stacked autoencoder (SSAE)-based deep neural network (DNN). The fluctuations and random amplitudes caused by multiple crack sizes cannot be overcome by analyzing the signal in just the time or frequency domain. However, a hybrid feature pool that is constructed after analyzing the vibration signal in different domains can provide sufficient information to effectively segregate bearings of different health conditions. Sparse stacked autoencoders (SSAEs) are deep neural networks (DNNs) that can extract intrinsic information from the input hybrid feature pool effectively, due to the highly nonlinear activation function used in the hidden layers. The first layer of the proposed scheme is for fault pattern identification, whereas the second layer identifies the crack size in each fault type.

The rest of the paper is organized as follows: Section 2 presents the proposed methodology. Section 3 describes the data set used for the experiments. Section 4 details the experimental results of the proposed scheme, and Section 5 concludes the paper.

## 2. Methodology

**Figure 1:**
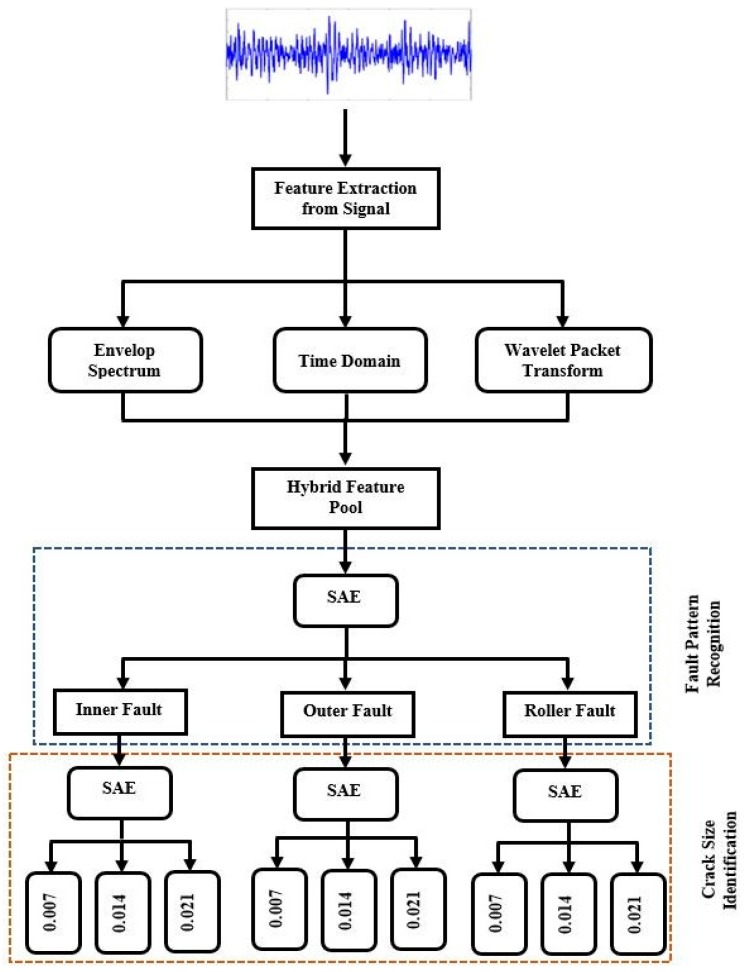
The proposed hierarchical fault diagnosis model, where SAE stands for stacked autoencoder.

The workflow of the proposed scheme is presented in Figure 1. The scheme can be divided into three phases. The first phase consists of hybrid feature pool generation, which involves combining time domain features, envelope power spectrum features, and wavelet energy features. In the next phase, the hybrid feature pool is provided as input to the stacked autoencoders to perform fault pattern identification (i.e., identifying inner raceway, outer raceway, and roller element faults). The last phase of the pipeline is to predict the crack size for a given fault.

The main idea of the work is to utilize a hybrid feature pool in combination with an SSAE-based DNN to extract high-level representative features, which would enhance the performance of the fault diagnosis model in the presence of multiple crack sizes. The hybrid feature pool provides more discriminating information about the raw vibrational signals and can overcome the nonstationary behavior of the input signal to boost the performance of the subsequent SSAE-based DNN. To create the hybrid feature pool, various feature extraction paradigms, including envelope power spectrum analysis, time domain analysis, and wavelet packet energy features, are used together.

### 2.1. Statistical Features 

The representative set of statistical time domain features used in [41] was adopted in our study. The time domain statistical features that are included in the hybrid feature pool are the root mean square value (*RMS*), kurtosis value (*KV*), square root of the magnitude (*SRM*), peak-to-peak value (*PPV*), standard deviation (*SD*), skewness value (*SV*), margin factor (MF), crest factor (*CF*), impulse factor (*IF*), kurtosis factor (*KF*), and mean value (*MV*). The given representative features are listed in Table 1 with their respective mathematical formulations.

### 2.2. Envelope Power Spectrum

A typical bearing found in a motor has four components: the outer raceway (OR), inner raceway (IR), cage (C), and the rolling elements (RE). At a constant speed, when a bearing has a defect on any of these components, periodic vibrations are generated. There are four fundamental defect frequencies: the ball spin frequency ($B_{SF}$), the ball pass outer raceway frequency ($B_{PFO}$), the ball pass inner raceway frequency ($B_{PFI}$), and the cage frequency ($F_{C}$). According to [42], the $B_{PFI}$, $B_{PFO}$, and $B_{SF}$ can be mathematically formulated as shown in Equations (1)–(3), respectively:

(1) $$B_{PFI}=\frac{N_{b}S}{2}\left(1+\frac{B_{d}}{P_{d}}\cos\phi \right),$$

(2) $$B_{PFO}=\frac{N_{b}S}{2}\left(1-\frac{B_{d}}{P_{d}}\cos\phi \right),$$

(3) $$B_{SF}=\frac{P_{d}}{2B_{d}}S\left[1-{\left(\frac{B_{d}}{P_{d}}\cos\phi \right)}^{2}\right].$$

Theoretically, if the defect is on the inner or outer raceway, an impulse is added to the vibration signal whenever the rolling element strikes the defective component. These impulses can be visualized from the associated defect frequencies, i.e., $B_{PFI}$ and $B_{PFO}$, respectively. If the defect is on a rolling ball, each time it strikes the inner raceway or outer raceway an impulse will be generated; theoretically, this will be twice the $B_{SF}$. These fundamental defect frequencies can be useful for identifying faults on the inner raceway, outer raceway, and the rolling element. These impulses at the associated defect frequencies can be explored via envelope spectrum analysis.

**Figure 2:**
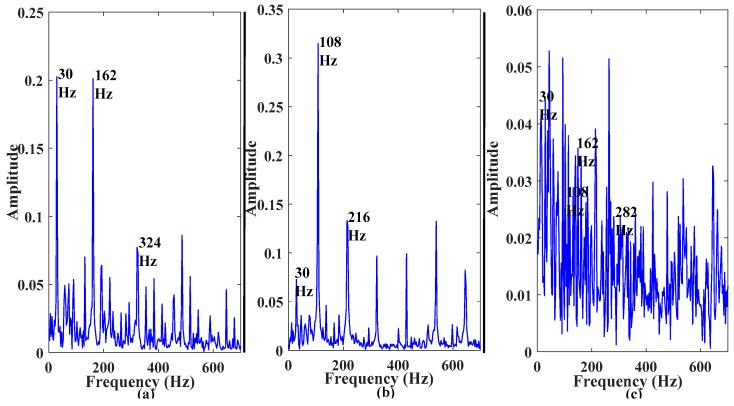
Envelope power spectrum: (**a**) inner raceway fault; (**b**) outer raceway fault, and (**c**) roller element fault.

The envelope of a vibration signal s(t) can be calculated by using the Hilbert transform. The Hilbert transform is a convolution between the Hilbert transform operator $\frac{1}{\pi t}$ and the original signal s(t) [43]. It can be represented as

(4) $$H\left[s(t)\right]=s(t)•\frac{1}{\pi t},$$

(5) $$H\left[s(t)\right]=\frac{1}{\pi }\int_{-\infty }^{\infty }\frac{s(t)}{t-\tau }dt,$$

where • is the convolution operator in (4) and H[s(t)] is an analytical signal of the original signal s(t). By taking the square of the fast Fourier transform of abs(H[s(t)]), a one-sided spectrum in the frequency domain can be obtained; this is the desired envelope power spectrum. The envelope power spectra of three fault types can be seen in Figure 2. Features extracted from the envelope spectra of the given example are presented in Table 2.

### 2.3. Wavelet Packet Transform (WPT)

**Figure 3:**
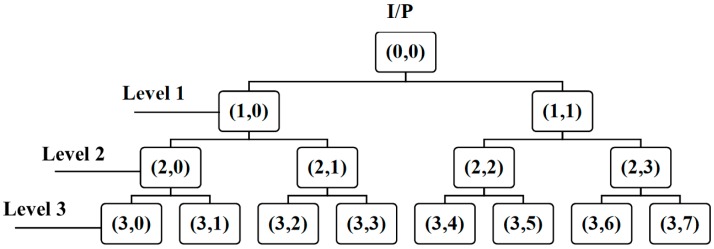
An illustration of a three-level wavelet packet tree decomposition, where I/P stands for input.

The wavelet packet transform (WPT) is a variation of the basic wavelet transform (WT) that decomposes the input signal into j levels. The WPT splits both the high-pass and low-pass filters, creating $2^{j}$ nodes at each level. The WPT overcomes the poor resolution of the WT by providing comprehensive time–frequency analysis of the signal at both low and high frequencies. Each level of the WPT provides a frequency range that is half as wide as the preceding level and twice as wide as the proceeding level. A three-level WPT tree structure can be seen in Figure 3.

The WPT coefficients can be formulated as

(6) $$c_{j+1}^{2k}(n)=c_{j}^{k}\times h(-2n),\;0<k<2^{j}-1,$$

(7) $$d_{j+1}^{2k+1}(n)=d_{j}^{k}(n)\times g(-2n),\;0<k<2^{j}-1,$$

where *h* and *g* are the low-pass and high-pass filters associated with the mother wavelet, respectively. These are predefined scaling factors. In the WPT, the scale parameter (level) is represented by *j*, and the frequency parameters (nodes) are represented by 2k and 2k+1.

Existing methods based on the WPT for bearing fault diagnosis consider the entropy, standard variation, and energy as input features to the subsequent classifier. Among these, using the wavelet packet energy is an intuitive approach to differentiating the fault types. The WPT nodes contain an abundance of information about the fault types and energy fluctuations in a specific node and can be useful in specifying the fault type.

In the current work, signals are decomposed up to j=4 levels, as described in [44], which results in $2^{j}=2^{4}=16$ nodes. After decomposition of the signals into different sub-bands, the WPT energy is computed by

(8) $$E=(\frac{\sum_{p=1}^{M}{\left(c_{j}^{k}{\left(p\right)}^{2}\right)}^{\frac{1}{2}}}{M}).$$

In the equation above, M is the number of samples at the nodes. All the energies acquired from j=4 level nodes are combined to form the vector V, which can be given as

(9) $$V=[E_{j}^{1},E_{j}^{2},...,E_{j}^{2^{j}}].$$

**Figure 4:**
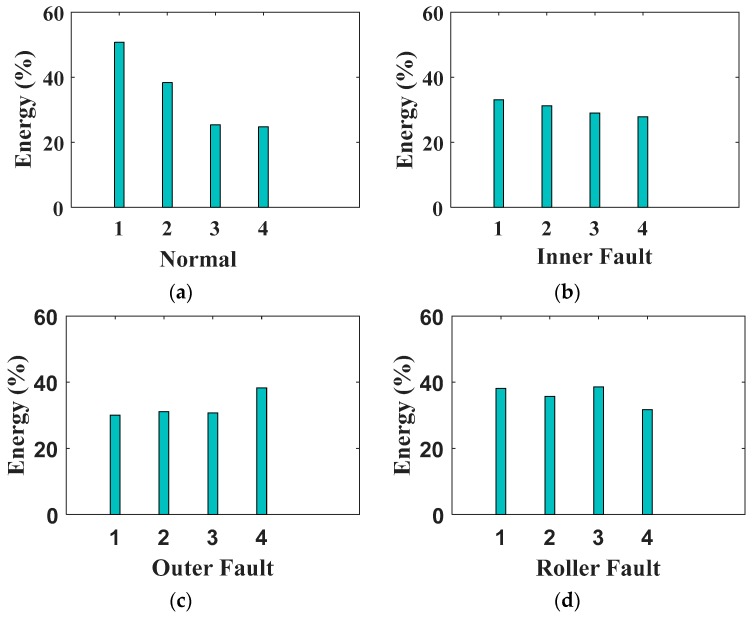
Wavelet energy features, (**a**) baseline condition; (**b**) inner fault signals; (**c**) outer fault signals; and (**d**) roller fault signals.

The maximum value of the vector is selected for each input signal and included in the hybrid feature pool. The extracted wavelet energy features can be seen in Figure 4. In the figure, wavelet energies for four different health conditions of the bearing are given. For each health condition, four signals are available—one for each motor load and rotational speed (i.e., 1722 to 1797 r/min). We notice that there is a variation in the wavelet energy levels for different health conditions, which can be of benefit to SSAEs in learning distinctive high-level features for a given health condition. On the other hand, there is also variation within the wavelet energy levels of a specific health condition, which can lead to confusion among the instances of different health conditions and can result in misclassification of the instances. To minimize the misclassification of the instances due to the variation in either the values of statistical features from the time domain, envelope power spectrum, or wavelet energy levels, a hybrid feature pool is formed by including the extracted time domain statistical features, envelope power spectrum, and WPT energy features. The hybrid feature pool can provide detailed intrinsic information about the nonstationary and nonlinear signals obtained from bearings with multiple fault severities. The length of the resulting hybrid features’ vector is 6+6+1=13. After creating the hybrid feature pool, it is provided as an input to the SAE-based DNN to learn high-level representative features and perform fault pattern recognition and fault severity classification.

### 2.4. Sparse Stacked Autoencoders (SSAEs)

**Figure 5:**
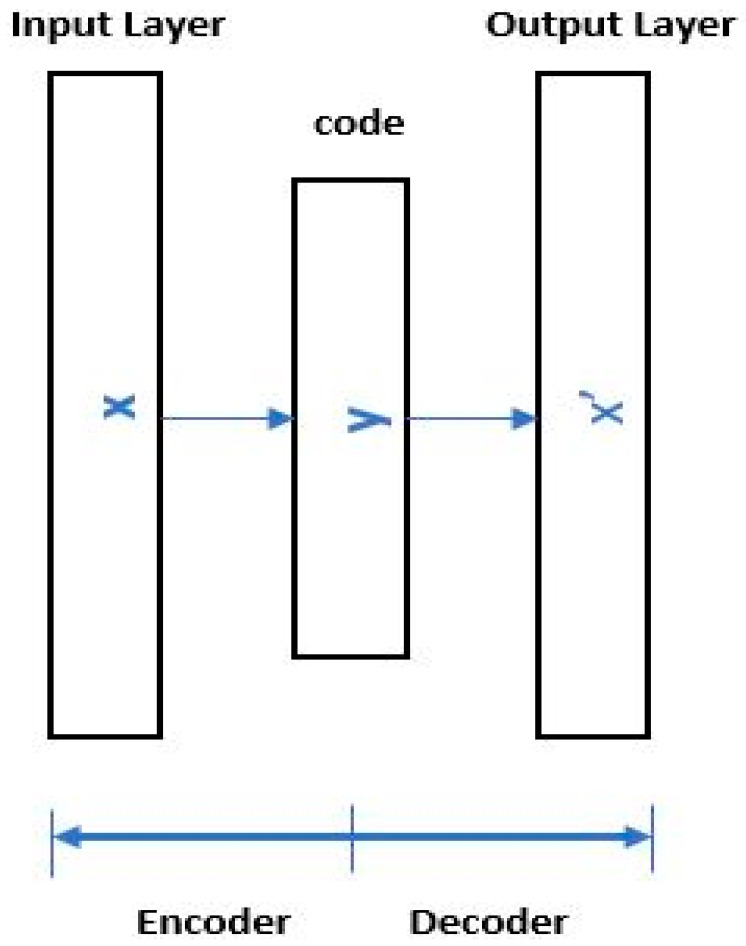
Structure of a basic autoencoder.

A simple autoencoder is basically a variation of an artificial neural network (ANN) with a minimum of three layers that uses an unsupervised learning process. The structure of a basic autoencoder is presented in Figure 5.

The first layer of the autoencoder is the input layer, which receives the input data. The intermediate layer tends to extract high-level representative features (i.e., latent codes) from the input data. The latent codes are, in essence, the result of principal component analysis (PCA) of the inputs and reduce the representation of the original data. The dimensionality of the latent codes depends on the number of nodes used in the hidden layer. The last layer decodes the latent codes and tries to reconstruct the original input. In short, an autoencoder performs two key tasks: to encode the input data into latent codes and then reconstruct the data from the latent codes. The resulting latent codes have lower dimensionality than the input data. In this regard, an autoencoder contributes to dimensionality reduction. The encoding ∂, and decoding β processes of an autoencoder are described as follows:

(10) $$\begin{array}{l} \partial :s\to F\\ \beta :F\to s\\ \partial ,\beta =\underset{\partial ,\beta }{\arg\min}{\left\|s-(\beta •\alpha )s\right\|}^{2}. \end{array}$$

The simplest form of an autoencoder has one hidden layer. The encoder stage receives input data s with dimension $R^{m}$ and maps the data to latent variables o with dimension $R^{n}$. The latent code can be given by

(11) o=g(Ws+b),

where o, W, b, and g are the latent code, weight matrix, bias vector, and activation function, respectively. Equation (12) presents the decoding process of an autoencoder:

(12) $$r=g^{\prime }(W^{\prime }s+b^{\prime })$$

where r, $W^{\prime }$, $b^{\prime }$, and $g^{\prime }$ are the reconstructed output, weight matrix, bias vector, and activation function of the decoder, respectively. The loss function is calculated between the original data and the reconstructed data in basic autoencoders by using the following loss function:

(13) $$L(s,r)=\frac{1}{M}\sum_{m=1}^{M}\sum_{k=1}^{K}{\left(s_{km}-r\right)}^{2}$$

where L is the loss calculated between the original data s and the reconstructed data r. A sparsity constraint can be introduced in an autoencoder by introducing a sparsity regularization term to the loss function. The sparsity constraint enables the autoencoder to learn useful features that can be used for classification [45]. The modified loss function can be represented as follows:

(14) $$L(s,r)=\frac{1}{M}\sum_{m=1}^{M}\sum_{k=1}^{K}{\left(s_{km}-r\right)}^{2}+\lambda \cdot \Omega_{weights}+\lambda^{\prime }\cdot \Omega_{sparsity}$$

where λ is the $L_{2}$ regularization coefficient and $\lambda^{\prime }$ is the sparsity regularization coefficient. $\Omega_{weights}$ is the $L_{2}$ regularization term and $\Omega_{sparsity}$ is the sparsity regularization term. $L_{2}$ regularization and $\Omega_{sparsity}$ regularization help in avoiding the overfitting problem in sparse autoencoders.

## 3. Dataset

**Figure 6:**
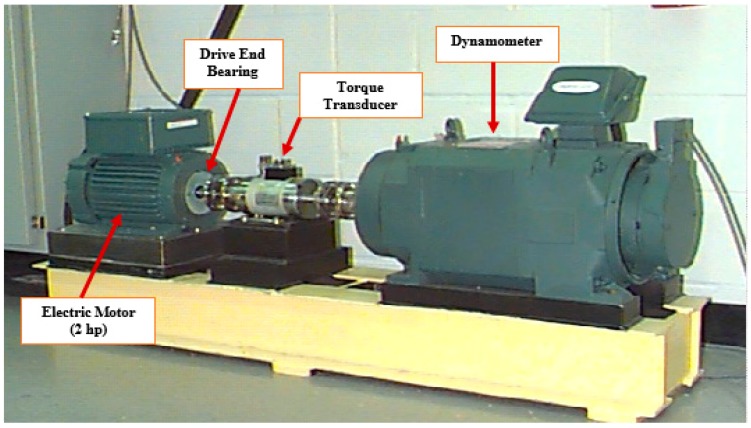
Case Western Reserve University’s seeded fault bearing testbed [46].

To demonstrate the efficacy of the proposed model, seeded fault data provided by Case Western Reserve University was used. As illustrated in Figure 6, the main components of the seeded fault test rig include a 2 horsepower (hp) electric motor, a dynamometer, and a torque transducer [46].

Using an electro-discharge machine, faults with diameters of 0.007, 0.014, and 0.021 inches were seeded on the inner raceway (IR), outer raceway (OR), and rolling elements (RE) at the drive end bearings. Variable length vibration acceleration signals were collected via an accelerometer attached to the housing of the drive end bearing at 12 o’clock with a sampling data rate of 12,000 Hz. The motor was subject to four loads ranging from 0 to 3 horsepower (hp), which resulted in four motor speeds, approximately from 1722 to 1797 revolutions per minute (r/min).

In this study, the dataset comprises vibration acceleration signals for normal bearings and bearings with three types of faults, i.e., faults on the inner raceway, outer raceway, and rolling element. For each fault condition, the dataset consists of signals recorded for bearings with three levels of fault severities (i.e., 0.007, 0.014, and 0.021 inches) at four different shaft loads. For normal bearings, there are four signals in the dataset—one for each shaft load. The signals are subjected to a segmentation process using a fixed sized window of 12,000 data points. The segmentation process splits all the fault signals into 10 samples each, but three of the four normal signals yield 20 samples each, while the fourth normal signal yields only 10 samples. The length of each sample for both normal and faulty bearings is 12,000 data points. Thus, the seeded fault dataset used for the experiments contains a total of 610 samples (70 normal samples + 3 fault types × 3 fault severities × 60 samples). After segmentation, a hybrid feature vector is constructed for each sample in the dataset. These feature vectors are then divided into training and test sets. The training set contains feature vectors for 310 samples (40 normal samples + 3 fault types × 3 fault severities × 30 samples), while the test set consists of feature vectors for 300 samples (30 normal samples + 3 fault types × 3 fault severities × 30 samples). The details of the bearing dataset with seeded faults are given in Table 3.

## 4. Results and Analysis

**Figure 7:**
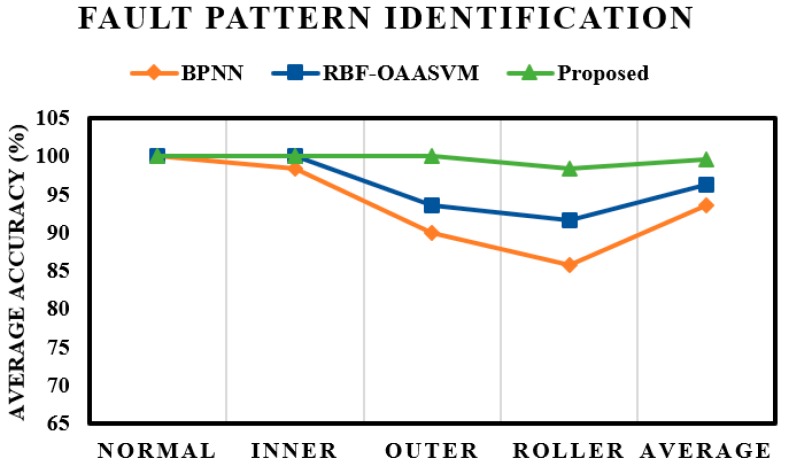
Bearing fault classification results for the first layer (i.e., the fault pattern recognition layer), BPNN and RBF-OAASVM stand for back-propagation neural network and radial basis function-one against all support vector machine, respectively.

A bearing dataset with seeded faults was provided by Case Western Reserve University [46] and used to validate the proposed fault diagnosis model. The dataset is composed of four health conditions and three different fault severities. For training and evaluation of the first layer, all the samples from training set were used to train the first sparse stacked autoencoder (SSAE)-based deep neural network (DNN). On the other hand, while training the rest of the three SSAE-DNNs in the crack size identification layer, only samples from the respective fault classes were considered. To produce stable results, the experiment was repeated 20 times with random selection of samples to form the train and test sets each time. To evaluate the effectiveness of the proposed scheme, the results were compared with those of the state-of-the-art algorithms, including the radial basis function (RBF) kernel-based one-against-all support vector machines (OAASVMs) and backpropagation neural networks (BPNNs). All the SSAE-DNNs in the proposed scheme were replaced with RBF-OAASVMs and BPNNs to create a similar hierarchical structure. The same set of features were provided as input to the RBF-OAASVMs and two layered BPNNs with 10 hidden nodes. The Levenberg–Marquardt backpropagation optimization function was used in the BPNNs to update the weights. Figure 7 presents the results of the fault pattern identification that is proposed to identify the bearing health conditions (i.e., normal health or having a fault on the inner raceway, outer raceway, or roller element) for the proposed method and the state-of-the-art algorithms. The overall average accuracy of the proposed model for the fault pattern identification layer is 99.5%. 

**Figure 8:**
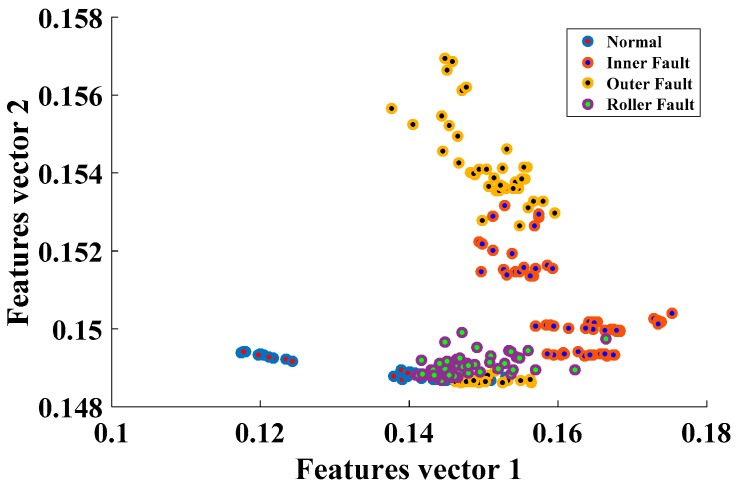
The extracted high-level features for the fault pattern recognition layer.

The SSAE-based DNN, because of its hierarchical structure and by using nonlinear transformation in the hidden layers, could extract discriminating information from the hybrid feature pool, enhancing the overall performance of the proposed model. This observation is validated by Figure 8, which contains the distribution of the first two feature vectors extracted by using SSAEs. It is worth noticing that the proposed method correctly classified all the samples for inner and outer faults; however, it misclassified a few of the roller fault samples. The hybrid feature pool fails to provide enough intrinsic information, and, thus, SSAEs fail to extract more discriminant high-level features in this case. In the case of the normal condition and inner and outer faults, high-level feature extraction seems relatively easy for SSAEs. In the case of roller fault, the extracted high-level features overlap with some of the samples from the normal and inner fault, which leads to the misclassification of roller fault samples. The roller fault signals possess the properties of inner as well as outer faults. This observation is validated by Figure 2, where the envelope power spectrum of the roller fault is given. The presence of inner and outer fault defect frequencies can be clearly seen in the envelope power spectrum. Therefore, features extracted from the time domain, envelope spectrum, and wavelet energy, in this case, were either confused with inner or roller fault. Moreover, from the comparison results, it is evident that the proposed model provided 3.32% and 6.12% better average accuracy for the fault pattern identification layer than the RBF-OAASVMs and BPNNs, respectively.

**Figure 9:**
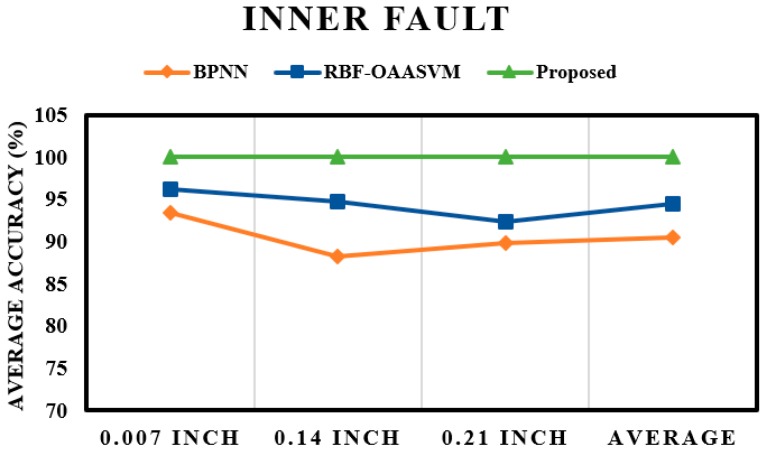
Fault severity prediction in an inner fault.

**Figure 10:**
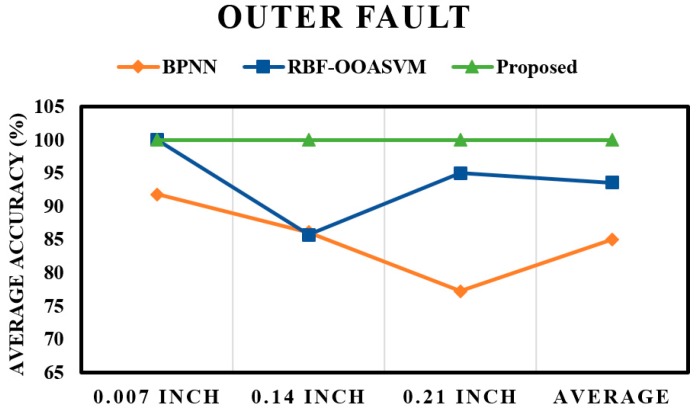
Fault severity prediction in an outer fault.

**Figure 11:**
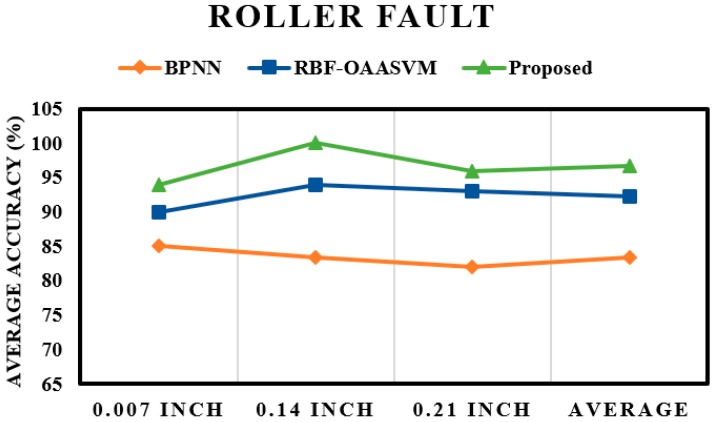
Fault severity prediction in a roller fault.

The fault pattern recognition layer is followed by the crack size identification layer. The results of the subsequent layer mainly depend on the results of the first layer; if the performance of the first layer is poor, the results of the subsequent layer will also be poor. From the results of the pattern recognition layer, it is evident that the proposed method could classify most of the input instances, which ultimately boosted the performance of the proposed scheme. This observation is validated by the results of the crack size identification layer. Figure 9 shows the fault severity classification of an inner fault; once again, the performance of the proposed method is better than those of the RBF-OAASVM and BPNN methods. The proposed method provides an average accuracy of 100%, whereas RBF-OAASVMs and BPNNs provide average accuracies of 94.4% and 90.44%, respectively. It can be interpreted from the results that the proposed scheme successfully classifies all the samples into their respective classes. However, RBF-OAASVMs and BPNNs fail to classify all the samples properly. Figure 10 shows the results for crack size prediction within an outer fault. The average fault severity accuracies for the proposed method, RBF-OAASVMs, and BPNNs are 100%, 93.56%, and 85.03%, respectively. In Figure 11, the results of crack size identification in terms of the average accuracy in a roller fault are given. It is evident that the proposed method outperforms the SVM and BPNN methods. In this case, there is slight deterioration in the performance of the proposed method, but, still, it delivers better performance compared with the other state-of-the-art algorithms. The deterioration is due to the misclassification of outer fault samples in the fault pattern identification layer, consequently affecting the results of the crack size identification layer in the case of roller faults. Overall, our proposed method has an average accuracy of 96.66%, while the average crack size prediction accuracies of SVMs and BNNs are 92.33% and 83.44%, respectively.

To further validate the reliability of the proposed method, a comparison is made with an existing bearing fault diagnosis scheme [47], in which the authors used vibration spectrum imaging (VSI) and artificial neural networks (ANN) for bearing fault diagnosis. The bearing dataset used for validation of the scheme was acquired from Case Western Reserve University (shaft load of 2 hp with 1748 r/min). The vibration signals were segmented into fixed sized windows of 1024 data points each, and then a 513 point fast Fourier transform (FFT) was applied to the segmented signals. The resultant spectral information was stacked on top of each other to generate a 513 × 8 pixel grayscale vibration spectrum image. A smoothing filter of size 8 × 4 was applied to the grayscale image, and then the filtered image was converted into a binary image by using an optimum threshold value of 0.7. The optimum threshold value plays a key role in the VSI-based fault diagnosis scheme because it defines the quality of the input vectors to the underlying classifier, and can affect the overall accuracy of the scheme. Then, the binary images, each with 4104 binary spectral components, were provided as an input to an artificial neural network having one hidden layer with three nodes. The comparison results of the proposed method and the VSI-based fault diagnosis scheme are presented in Table 4. The proposed method provides better diagnostic performance as compared with VSI when tested on the dataset containing instances from the seeded fault bearings with multiple fault severities. The proposed method can overcome the nonstationary and nonlinear behavior of the vibration signal in a much better way compared with the VSI-based approach, where the spectral information is more susceptible to variation in working conditions and fault severities.

## 5. Conclusions

In this paper, a two-layered bearing fault diagnosis scheme was proposed. The first layer is for fault pattern identification in rotary machine bearings, while the subsequent layer is used for crack size identification of a given fault. A hybrid features pool comprising time domain statistical features, envelope power spectrum features, and wavelet energy features is used in combination with sparse stacked autoencoder (SSAE)-based deep neural networks (DNNs) for the diagnosis of different bearing defects with various levels of severity. The hybrid feature pool was formed to overcome the nonstationary and nonlinear behavior of the vibration acceleration signals. A bearing dataset containing four health conditions and three fault severities was used to validate the proposed model. It is observed that the SSAE-based DNN is able to extract effective representative features from the hybrid feature pool, resulting in a superior diagnostic performance of the proposed model for both fault pattern as well as for crack size identification. Moreover, the proposed model was compared with three state-of-the-art fault diagnosis algorithms (i.e., RBF-OAASVMs, BPNNs, and VSI). The results demonstrated that the proposed scheme is more effective compared with the other methods regardless of the nonlinearity contained in the vibration signals due to multiple fault severities. However, in the case of roller fault identification, the performance of the proposed method slightly deteriorated, which underscores the need for more sophisticated signal processing algorithms as future work that could eventually result in superior diagnostic performance. It can be concluded that the proposed method provides satisfactory bearing fault diagnosis results and can be used for fault diagnosis of bearings containing various fault severities.

## Figures and Tables

**Table 1:** Time domain statistical features (*x* is the vibrational signal).

Features	Equations	Features	Equations	Features	Equations
Mean value (*MV*)	$\bar{x}=\frac{1}{N}\sum_{i=1}^{N}x_{i}$	Standard deviation (*SD*)	$\sigma^{2}=\frac{1}{N-1}\sum_{i=1}^{N}{\left(x_{i}-\bar{x}\right)}^{2}$	Root mean square (*RMS*)	$RMS={\left(\frac{1}{N}\sum_{i=1}^{N}x_{i}^{2}\right)}^{\frac{1}{2}}$
Peak-to-peak value (*PPV*)	$PPV=\max(x_{i})-\min(x_{i})$	Skewness value (*SV*)	$SV=\frac{1}{N}\sum_{i=1}^{N}{\left(\frac{x_{i}-\bar{x}}{\sigma }\right)}^{3}$	Margin factor (*MF*)	$MF=\frac{\max(\left|x_{i}\right|)}{{\left(\frac{1}{N}\sum_{i=1}^{N}\sqrt{\left|x_{i}\right|}\right)}^{2}}$
Crest factor (*CF*)	$MF=\frac{\max(\left|x_{i}\right|)}{(\frac{1}{N}\sum_{i=1}^{N}x_{i}^{2})\frac{1}{2}}$	Impulse factor (*IF*)	$IF=\frac{\max(\left|x_{i}\right|)}{\frac{1}{N}\sum_{i=1}^{N}\left|x_{i}\right|}$	Square root of the magnitude (*SRM*)	$SRM=(\frac{1}{N}\sum_{i=1}^{N}\sqrt{\left|x_{i}\right|}{)}^{2}$
Kurtosis value (*KV*)	$KV=\frac{1}{N}\sum_{i=1}^{N}{\left(\frac{x_{i}-\bar{x}}{\sigma }\right)}^{4}$	Kurtosis factor (*KF*)	$KF=\frac{\frac{1}{N}\sum_{i=1}^{N}{\left(\frac{x_{i}-\bar{x}}{\sigma }\right)}^{4}}{(\frac{1}{N}\sum_{1}^{N}x_{i}^{2}{)}^{2}}$		

**Table 2:** Statistical features extracted from the envelope power spectrum.

Feature	Equation
**RMS frequency**	$RMS_{f}={\left(\frac{1}{K}\sum_{i=1}^{K}{y_{K}}^{2}\right)}^{\frac{1}{2}}$
**Frequency center**	$FC=\frac{1}{K}\sum_{i=1}^{K}y_{K}$
**Standard deviation**	$\sigma_{f}^{2}=\frac{1}{K}\sum_{i=1}^{K}{\left(y_{K}-FC\right)}^{2}$
**Root variance frequency**	$RVF={\left(\frac{1}{K}\sum_{i=1}^{K}{\left(y_{K}-FC\right)}^{2}\right)}^{\frac{1}{2}}$
**Spectral kurtosis**	$K_{f}=\frac{1}{K}\frac{\sum_{i=1}^{K}{\left(y_{K}-FC\right)}^{4}}{{\left(\sigma_{f}^{2}\right)}^{2}}$

**Table 3:** Bearings and dataset specification.

Fault Type	Fault Location	Fault Size (Inches)	Training Samples	Test Samples	Sample Length	Accelerometer Position	Shaft Load (hp)
Normal	None	0	40	30	12,000	Drive End Bearings	0, 1, 2, 3
Inner raceway	IR	0.007	30	30			
	IR	0.014	30	30			
	IR	0.021	30	30			
Outer raceway	OR	0.007	30	30			
	OR	0.014	30	30			
	OR	0.021	30	30			
Roller	RE	0.007	30	30			
	RE	0.014	30	30			
	RE	0.021	30	30			

**Table 4:** The diagnostic performance of the proposed model and vibration spectrum imaging (VSI).

Method	Layer 1 Average Accuracy (%)	Layer 2 Average Accuracy (%)			Total (%)
		0.007 Inches	0.014 Inches	0.021 Inches	
VSI [47]	60.15	55	55	84.6	63.68
Proposed	99.75	100	100	96.66	99.10
